# Holistic View on Cell Survival and DNA Damage: How Model-Based Data Analysis Supports Exploration of Dynamics in Biological Systems

**DOI:** 10.1155/2020/5972594

**Published:** 2020-07-06

**Authors:** Mathias S. Weyland, Pauline Thumser-Henner, Katarzyna J. Nytko, Carla Rohrer Bley, Simone Ulzega, Alke Petri-Fink, Marco Lattuada, Rudolf M. Füchslin, Stephan Scheidegger

**Affiliations:** ^1^Zurich University of Applied Sciences Winterthur, Switzerland; ^2^BioNanomaterials Group, Adolphe Merkle Institute, University of Fribourg, Fribourg, Switzerland; ^3^Division of Radiation Oncology, Vetsuisse Faculty, University of Zurich, Zurich, Switzerland; ^4^Center for Applied Biotechnology and Molecular Medicine (CABMM), University of Zurich, Zurich, Switzerland; ^5^Center for Clinical Studies, Vetsuisse Faculty, University of Zurich, Switzerland; ^6^Department of Chemistry, University of Fribourg, Fribourg, Switzerland

## Abstract

In this work, a method is established to calibrate a model that describes the basic dynamics of DNA damage and repair. The model can be used to extend planning for radiotherapy and hyperthermia in order to include the biological effects. In contrast to “syntactic” models (e.g., describing molecular kinetics), the model used here describes radiobiological semantics, resulting in a more powerful model but also in a far more challenging calibration. Model calibration is attempted from clonogenic assay data (doses of 0–6 Gy) and from time-resolved comet assay data obtained within 6 h after irradiation with 6 Gy. It is demonstrated that either of those two sources of information alone is insufficient for successful model calibration, and that both sources of information combined in a holistic approach are necessary to find viable model parameters. Approximate Bayesian computation (ABC) with simulated annealing is used for parameter search, revealing two aspects that are beneficial to resolving the calibration problem: (1) assessing posterior parameter distributions instead of point-estimates and (2) combining calibration runs from different assays by joining posterior distributions instead of running a single calibration run with a combined, computationally very expensive objective function.

## 1. Introduction

DNA damage and repair is a critical aspect of radiotherapy, where tumor cells are killed by irradiation. The radiation induces DNA damage which eventually leads to cell death if the damage cannot be repaired successfully. Mild hyperthermia is a treatment to boost radiotherapy by heating up the cancer cells to temperatures between 41°C and 43°C. While the exact working principles of hyperthermia and its interaction with radiotherapy is still subject to research [1], it has been shown that hyperthermia acts as a radiosensitizer by affecting the DNA repair that takes place after an irradiation event [2–5]. In consequence, knowledge about the dynamics of DNA damage and repair is essential in order to optimize hyperthermia treatment plans.

For radiotherapy, in silico modeling is employed to assist in treatment planning decisions. Such planning is based on Monte Carlo simulations or kernel methods and deliver dose-volume histograms [6]. Beyond these geometric dose calculations, approaches to shape the prescribed radiation dose according to the biological properties of the tumor have been proposed but are currently not established [7]. The prescribed dose of radiation is generally divided into fractions that are delivered in subsequent sessions; however, this fractionation scheme is usually not optimized on a patient level, and the dose prescription is chosen based on clinical trials and experience. While planning software may include calculators for biological effective dose (BED) and equivalent dose (EQD2), they are not modeling biological effects (such as DNA damage and repair), but rather, they are tools for comparing fractionation schemes. Similarly, for hyperthermia, planning systems for hyperthermia output temperature or specific absorption rate (SAR) maps exist [8] and calculators for equivalent doses have been proposed [9, 10]. Yet, a more profound understanding and modeling of the aforementioned radiobiological effects—DNA damage and repair in this context—would yield a better treatment method. For example, hyperthermia is believed to deactivate DNA repair proteins for a certain amount of time [2]. If radiation-induced damage is introduced during this time window, odds of eliminating the cells increase [11]. Thus, if calibrated correctly, a model involving DNA damage and repair would be able to quantify the duration of this window by simulating the de- and reactivation of said proteins.

In this work, a method is established to calibrate a model that describes the basic dynamics of DNA damage and repair. This model can then be used to extend planning for radiotherapy and hyperthermia to include the biological effects discussed above, i.e., DNA damage and repair: The biological system is modeled in silico, and a parameter search for model calibration is performed with the goal to be able to quantify biological effects for the system of interest. While previous efforts demonstrated feasibility [12], a thorough analysis of the calibration process is provided. This analysis reveals that some parameters remain unidentified. One strength of the method is that it is able to combine calibration results originating from different input data sources (i.e., assays). With this approach, the yet unidentified parameters could be further refined.

**Figure 1:**
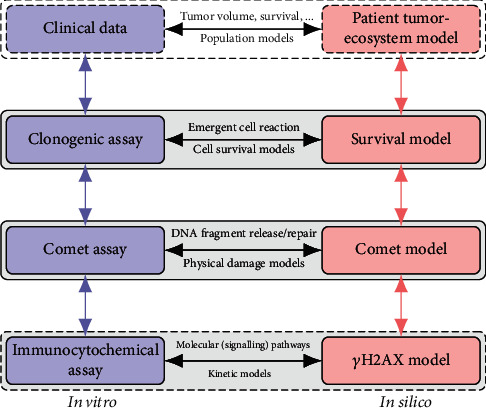
Overview of different assays (clonogenic, comet, and immunocytochemical) capturing different aspects of DNA damage and repair (cell survival, physical damage, and molecular pathways). Each assay (left) provides data that correspond with a suitable model in silico (right). Alternatively, those various aspects can be captured in a single, holistic model from which synthetic assay data are derived for comparison. The latter approach is pursued in this work for emergent cell reactions (clonogenic assay) and physical DNA fragment repair (comet assay).

Model calibration requires data which can be obtained from number sources which are shown in Figure 1: (1) immunocytochemical assays such as *γ*H2AX, which quantify DNA repair [13]; (2) comet assay, which quantifies the amount of DNA damage [14]; this assay is further discussed in Section 2.3; (3) clonogenic assay, which quantifies clonogenicity [15] and is discussed in Section 2.2. (4) In a clinical setting, DNA damage and repair in tumor cells also affects response evaluation criteria, tumor volume, patient survival, tumor progression and growth rate, etc. Thus, these data (yet quite heterogeneous [7] and thus potentially a poor choice) could, in theory, also be used for model calibration.

These four different options correspond to the four levels illustrated in Figure 1 on the left. On the right, suitable models for these types of readout are depicted. Often, these models merely attempt to replicate some observed readout. For example, the cell survival curves discussed above usually exhibit a parabolic nature in the logarithmic domain [16, 17]. Thus, a quadratic model for log(*S*) is often used for the dose-response, without further rationale but just as a method for fitting the existing data. In the past, this approach has been expanded to a linear-quadratic-linear relationship [18], again in a mere attempt to mimic experimentally observed data. Similarly, biostatistical models for comet assay analysis are able to describe the assay readout but do not model actual DNA damage and repair, let alone in a dynamic fashion [19, 20].

Another class of models go one step further and actually describe underlying molecular principles instead of the mere assay readout. For example, the H2AX phosphorylation discussed above can be modeled using a set of differential equations [21], and the *γ*H2AX readout is derived from the model. This approach is mechanistic in the sense that it is directly modeling the kinetics of the *γ*H2AX pathway and can be seen as syntactic description of molecular mechanisms. Other models including the lethal-potentially-lethal (LPL) model by Curtis [22] and the model by Vassiliev [23] and the Γ-LQ model [24] all follow radiobiologically motivated approaches but do not consider hyperthermia. The AlphaR model [25] takes the effect of hyperthermia into account, albeit for temperatures above 43.5°C which are not the focus of this work. Going one step further, the multi-hit-repair (MHR) model describes radiobiological semantics [26] instead of mechanics. It was used to derive cell survival curves [12] as well as comet assay readouts [27]. In addition to the semantic approach, the MHR model was chosen for this work because it is bioinspired and in the past, its ability was shown to explain many radiobiological phenomena.

## 2. Materials and Methods

In the following sections, the experimental setup (Section 2.1), the different biological assays (Section 2.2 and 2.3), the model used in this work (Section 2.4), the methods to map the model state to the readout from experimental assays (Section 2.5), and the calibration method (Section 2.6) are introduced, concluding with a brief section about the software and its availability (Section 2.7).

### 2.1. Experimental Setup

Hyperthermia and irradiation was performed on cells from the Abrams cell line; they were a kind gift of Prof. Robert Rebhun (University of California, Davis, California, USA). These canine osteosarcoma cells were selected because of their radioresistance (SF2: 0.85) [28], yet they respond well to hyperthermia as a radiosensitizer (*α* = 4.6 × 10^−3^ Gy ^−1^, *β* = 6.4 × 10^−3^ Gy ^−2^, and *α*/*β*=0.72 Gy with hyperthermia enhancement-factors (EF) *α*_EF_ = 6.7 and *β*_EF_ = 1.2 [29] for hyperthermia performed as indicated below.)

Cells were kept in DMEM at 37°C in a humidified incubator with 5% CO_2_ (MCO-18AC-PE, Sanyo, Osaka, Japan). In case of a hyperthermia treatment preceding irradiation, the cells were transferred to another incubator of the same type, set to 42°C, and exposed to a heat-up phase of approx. 40 min, followed by another 60 min of treatment time at the target temperature. The sequence of treatments (hyperthermia followed by irradiation) and the treatment time were chosen to match the clinical practice [30]. To ensure repeatability and quantify thermodynamic effects such as heat transfer and evaporative cooling, incubators were carefully calibrated [29]. In case of an experiment without hyperthermia treatment, the cells remained in the 37°C incubator. Upon completion of the hyperthermia treatment time, the cells were removed from the incubators and irradiated with a 6 MV linear accelerator (Clinac iX, Varian, Palo Alto, USA). Adequate dose build-up and optimal homogeneity of the dose distribution over the irradiation field were ensured by appropriate layers of Plexiglass. Since the irradiation device is also used for regular animal patient treatments, the dose calibration is carried out by a board-certified, qualified medical physicist and is regularly checked with an ionization chamber calibrated at the Swiss Federal Institute of Metrology (METAS).

**Figure 2:**
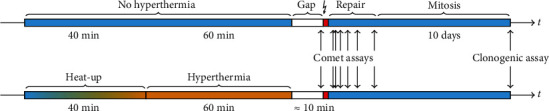
Graphical representation of the experimental treatment. The timeline at the top depicts an experiment without hyperthermia, where cells are kept at 37°C, then transferred to the linear accelerator, irradiated (↯), and then left to grow clones. Comet assays are performed prior to irradiation as well as during the ≈6 h of repair time post irradiation. For clonogenic assay, the clones are fixed and quantified after 10 days. The timeline at the bottom follows the same scheme, but with the additional hyperthermia treatment including the ramp-up to and treatment at 42°C. The time axis is not drawn to scale.

For logistic reasons (transfer time, setup time, and sequence of irradiation), there was a time-gap of approx. 10 min between the end of the hyperthermia treatment and the beginning of irradiation. Irradiation occurred at doses between 0 Gy and 6 Gy with a dose rate of 600 MU, corresponding to approx. 6 Gy/min.  Figure 2 illustrates the timeline of the experiments. It is important to note that while the timeline may suggest otherwise, any experimental procedure (clonogenic and comet assay) discussed below is destructive to the cells. Cells used for a given readout can therefore not be used again for a later or different readout. Thus, the readout originates from different batches of cells.

### 2.2. Clonogenic Assay

Clonogenic assay is a method to quantify the fraction of cells that survive a treatment, in this case an irradiation event [15]. It works by seeding a number of cells in a dish such that colonies form around these cells due to cell division. After 10 days, the number of colonies are counted and related to the number of cells seeded. If a cell loses clonogenicity due to the treatment, it will not form a clone, while cells which survive the treatment (in the sense of maintaining clonogenicity) will form a colony. The dataset to model clonogenic cell survival in canine osteosarcoma Abrams cells used here was previously published, and the details of the experimental protocol are described in [29].

### 2.3. Comet Assay

Comet assay is a method to quantify physical DNA damage in individual cells [14] and was performed as follows: approximately 1.5×10^5^ Abrams cells were seeded in each well of 6-well plates the day before treatments. Cells were treated with radiation and/or heat and harvested after treatments. For this, trypsin was used, and cells were then resuspended in ice-cold PBS. After centrifugation, cells were counted in each sample and resuspended in their DMEM culture medium complemented with 10% DMSO, in an appropriate volume to reach the concentration of 2×10^5^ cells per mL. Samples for cells used in comet assay were then stored at -80°C. Experiments were repeated 3 times.

Cells from every repeated experiment were thawed on the same day and run for comet assay (5 different runs were needed to run all the samples). After thawing and centrifugation, DMSO was quickly removed and ice-cold PBS added. Cells were suspended in molten LMAgarose (CometAssay® LMAgarose, Trevigen) at a ratio of 1/10 (approximately 1500 cells per sample). Cells were embedded in agarose on a glass slide and left in the dark for 10 min at 4°C. Slides were then immersed in a 4°C lysis solution (CometAssay® Lysis Solution, Trevigen) for 1.5 h in a room at 4°C. Slides were then immersed in the electrophoresis running buffer (8 mg/mL NaOH, 2 mL/mL 0.5 M EDTA pH 8, in dH_2_O) for 10 min at 4°C in the dark. For electrophoresis, slides were placed in the Trevigen Comet assay tank (CometAssay® Electrophoresis System II, Trevigen) in a cold room, in an exact volume of 850 mL of 4°C electrophoresis solution. Runs lasted 30 min at 21 V and 0.3 A. Care was taken to maintain the same temperature and volume of solution between runs to avoid interrun variability. Slides were finally immersed twice in dH _2_O for 10 min each, then in 70% ethanol for 15 min at room temperature. For staining, diluted SYBR Gold (1 : 10 000, SYBR Gold Nucleic Acid Gel Stain, Invitrogen) was then added to each spot of dried agarose including cells, for 15 min at room temperature, in the dark. Slides were rinsed, dried, and stored at room temperature in the dark.

In order to quantify DNA damage, the microscopy image of the stained comets is analysed with the image processing software COMET IV, which computes a value for each cell/comet, indicating the degree of DNA damage. From the damage metrics offered by the software, the relative tail intensity (RTI) was chosen because it provides a linear relationship between the number of DNA strand breaks and the quantified damage [31, 32]—a property highly desired for the data-mapping introduced in Section 2.5. The resulting data was used in a previous publication [27].

### 2.4. The Multi-Hit-Repair Model

**Figure 3:**
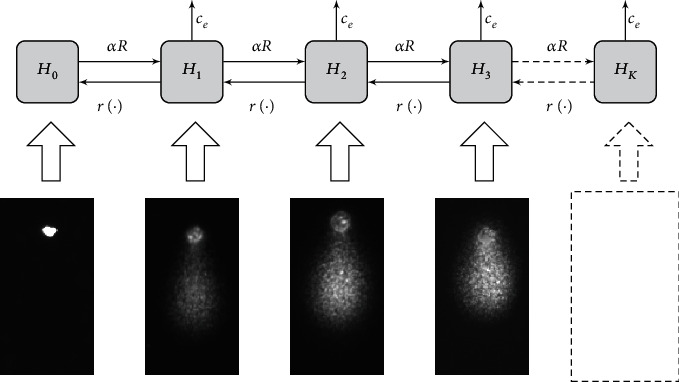
High-level illustration of the MHR model [27]. The boxes depict the chain structure with the populations *H*_*i*_; the arrows denote how cells accumulate hits (to the right), undergo cell death (to the top), or undergo repair (to the left). Below the chain, comet assay pictures conceptually illustrate how comets with increasingly high relative tail intensities are mapped to populations with increasingly high numbers of hits.

As mentioned in [27], the MHR model [12] is a dynamic population model where cells are assigned to populations *ℋ*_*i*_ depending on the number of radiation-induced hits (thus the variable name *ℋ*) they have accumulated. The variable *H*_*i*_ counts the number of cells in population *ℋ*_*i*_. A hit is defined in this work as a lesion that is hindering the cell from mitosis. In consequence, cells with one or more hits cannot undergo mitosis until all the hits are cured by the repair process. Clonogenicity is the ability of cells to form clones, for which mitosis is a prerequisite. Thus, only the cells in *ℋ*_0_ are clonogenic.  Figure 3 provides a graphical illustration of the model. Cells can accumulate up to *K* hits, corresponding to the length of the aforementioned chain. The chain length could be infinite, but for an implementation, *K* has to be limited. The practical limit for *K* is chosen such that no congestion at the end of the chain occurs. This criterion is met at *K* = 9; thus, the chain length was chosen accordingly.

During a simulation run, all cells are clonogenic at first; thus, they are assigned to population *ℋ*_0_, counted by the state variable *H*_0_. Hits are induced by radiation with dose rate *R*(*t*), which is set to 0 Gy/min at any time except during irradiation. Thus, *R*(*t*) is a square pulse that starts at *t* = 0 with an intensity of 6 Gy/min (see Section 2.1); the width of the pulse corresponds to the administered dose. While *R*(*t*) > 0, cells conceptually travel into the chain as they accumulate hits according to a radiosensitivity parameter *α* (*α* in the context of the MHR model is unrelated to *α* as used in the linear-quadratic model mentioned in Introduction). After irradiation, repair processes inside the cells cure the lesions and thus, cells travel in the opposite direction where they eventually may reach *ℋ*_0_. This repair is governed by the repair rate constant *c*_*r*_ and modulated by a repair function *r*(·) (see below). Alternatively, the repair processes may fail, leading to the death of a cell. This elimination process occurs at a rate of *c*_*e*_*H*_*i*_. Thus, the differential equations for population *ℋ*_*i*_ is

(1) $$\begin{array}{c} \frac{\text{d}H_{i}}{\text{d}t}=\alpha R\left(t\right)H_{i-1}-\alpha R\left(t\right)H_{i}-r\left(H_{i}\right)+r\left(H_{i+1}\right)-c_{e}H_{i}. \end{array}$$

DNA repair cannot occur immediately after repair, since radiation not only induces DNA damage but also damages the proteins required for repair. The consequent initial impedance of repair is modeled using the transient biological dose equivalent (TBDE) Γ:

(2) $$\begin{array}{c} \frac{\text{d}\Gamma }{\text{d}t}=R\left(t\right)-\gamma \Gamma . \end{array}$$

Γ decays after irradiation and is used in the repair function to impede repair after irradiation:

(3) $$\begin{array}{c} r\left(H_{i}\right)=c_{r}\exp\left(-\mu_{\Gamma }\Gamma \right)H_{i}. \end{array}$$

Since some small amount of damage is already present before irradiation, initial conditions were chosen to reflect the damage distribution according to Equation (8) in prior work [27]. Alternatively, it can be assumed that no damage is present before irradiation; i.e., *H*_0_(0) = 1, *H*_*i*>0_(0) = 0, and Γ(0) = 0. Negligible differences in terms of the model output were found between these two approaches; thus, the latter, simpler approach is used in this work. The full set of equations is given in the supplementary materials; a summary of the model parameters is presented in Table 1. See [12, 26, 27] for the derivation, validation, and further discussion of the MHR model.

For hyperthermia, the two variables *Y* and *Λ* are introduced to track the state of active (*Y*) and inactive (*Λ*) repair proteins. These variables represent the respective relative amount of repair protein, and thus, they sum up to 1; i.e., *Y* + *Λ* = 1. The activation and inactivation is governed by the following differential equations:

(4) $$\begin{array}{c} \frac{\text{dY}}{\text{d}t}=-k_{1}Y+k_{2}\Lambda , \end{array}$$

(5) $$\begin{array}{c} \frac{\text{d}\Lambda }{\text{d}t}=k_{1}Y-k_{2}\Lambda . \end{array}$$

The rate at which inactive repair protein is reactivated, *k*_2_, is assumed to be constant in prior research [12, 26, 27, 30] and throughout this work. While the reactivation may be temperature-dependent, the authors are unaware of any research supporting that hypothesis, thus, following the principle of assuming simple circumstances whenever possible and, *k*_2_ is not a function of temperature.

The inactivation of repair protein, however, is temperature-dependent [2, 33, 34]. The rate at which this occurs, *k*_1_, incorporates the Arrhenius law [35] as follows:

(6) $$\begin{array}{c} k_{1}=a\cdot 10^{-3}\exp\left(\frac{E_{a}}{\bar{R}\left(273.16+37\right)}-\frac{E_{a}}{\bar{R}\left(273.16+T\right)}\right). \end{array}$$

The parameter *a* is introduced for numeric reasons. $\bar{R}=8.314$ J·K ^−1^·mol ^−1^ is the gas constant, and *E*_*a*_ = 1528 kJ·mol ^−1^ is the activation energy as published in the literature [12, 36]. It is important to note that *E*_*a*_ may be cell-line specific; thus, the choice of *E*_*a*_ should be revisited in the future once such data is available for the Abrams cell line used here. It is easy to show that the equilibrium of Equations (4) and (5) are *Y* ≈ 1 and *Λ* ≈ 0, respectively, for *T* = 37 °C. Those values therefore serve as initial conditions as it is assumed that this equilibrium is reached prior to the hyperthermia treatment.

The repair function is extended to modulate the DNA repair rate with the amount of inactive repair proteins:

(7) $$\begin{array}{c} r\left(H_{i}\right)=c_{r}\exp\left(-\mu_{\Gamma }\Gamma -\mu_{\Lambda }\Lambda \right)H_{i}. \end{array}$$

This entails that the rate of repair is reduced both in the presence of inactive repair protein due to thermal effects (*Λ*) and after irradiation when the TBDE is high (Γ).

A temperature of *T* = 42°C is set during the hyperthermia treatment. Before and after the treatment, the temperature is set to *T* = 37°C.

### 2.5. Model/Readout Mapping

Since the MHR model is describing radiobiological processes instead of assay readouts, methods need to be implemented to map the model to such readouts. For clonogenic assay, this is relatively straight forward and was introduced in [12]: *ℋ*_0_ is tracking clonogenic cells by definition; thus, the surviving number of cells is readily available in *H*_0_. Survival *S* is therefore found by evaluating *H*_0_ at the end of the simulation, provided the simulation time is chosen such that the repair process has completed at the end of the simulation.

The mapping to comet data is somewhat more elaborate and was introduced in [27]: The comet readout at a given point in time consists of the quantification of DNA damage in a number of (typically *m* = 100) cells. Depending on the amount of DNA damage, each cell is assigned to a bin *h*_*i*_: the first bin *h*_0_ tracks the cells with little to no damage and the second bin *h*_1_ tracks cells with more damage, etc. The cell count in each bin and population is normalized such that

(8) $$\begin{array}{c} \overset{K}{\underset{i=0}{\sum }}\;{\tilde{h}}_{i}=\overset{K}{\underset{i=0}{\sum }}\;{\tilde{H}}_{i}=1. \end{array}$$

Finally, the relative bins ${\tilde{h}}_{i}$ can be mapped directly to ${\tilde{H}}_{i}$ of the MHR model.

In Section 2.4, a hit was defined as an impact on the cell that bars it from mitosis until cured. The correct mapping between physical DNA damage as reported by the comet assay and the model populations *ℋ*_*i*_ presumes knowledge about how much physical DNA damage constitutes one hit. In other words, the relative tail intensities quantifying DNA damage must be scaled prior to the mapping to *H*_*i*_ to maintain the semantics implied by the MHR model (i.e., the definition of a hit). In [27], the correct scaling factor was unknown, and thus, tail intensities between 0 and 4% were mapped to *ℋ*_0_ arbitrarily (as discussed there, the model can still be used with a wrong scaling factor, but the parameters may lose the meaning they were originally designed for). In this work, the scaling factor is not fixed to a single, convenient value arbitrarily. Instead, the procedure is repeated with different scaling factors within a sensible range. In order to achieve this, the scaling is formalized by the variable *σ* which denotes the largest tail intensity that is still mapped to *ℋ*_0_. Hence, *σ* = 0.04 in the above example.

Interestingly, the method failed to reproduce experimental comet data for small values of *σ*. For large values of *σ*, the resulting *α* parameter values were in violation of the lower bound stipulated by Equation (9) (see Figure S1). Only a small region around *σ* = 0.03 was free from these issues; thus, *σ* = 0.03 was used.

### 2.6. Approximate Bayesian Computation

**Figure 4:**
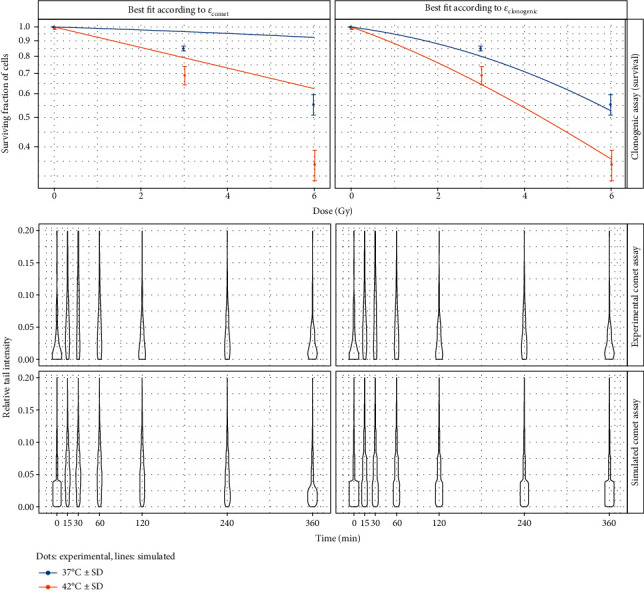
Top: cell survival curves after parameter search with Equation (11) as objective function: *α* = 0.19, *c*_*r*_ = 0.22, *c*_*e*_ = 0.00, *μ*_Γ_ = 0.00, *γ* = 3.80, *a* = 1.00, *k*_2_ = 0.01, and *μ*_*Λ*_ = 3.50 (left); *α* = 0.23, *c*_*r*_ = 5.80, *c*_*e*_ = 0.14, *μ*_Γ_ = 1.62, *γ* = 0.26, *a* = 0.31, *k*_2_ = 1.3 · 10^−4^, and *μ*_*Λ*_ = 4.67 (right). The plots at the left result from a parameter search where clonogenic assay data is not considered at all (i.e., comet data only as published and discussed in [27]). The plots at the right also show results from a parameter search from comet data alone, but the parameter set producing the best clonogenic cell survival curve (according to Equation (10)) is shown. Since information from clonogenic assay was used, the left plot exhibits a very poor prediction of experimental data (*ε*_clonogenic_ = 0.12). This shows that the data from comet assay alone do not capture all information required for a successful parameter search. However, some parameter sets are viable—the plot at the right does not suffer from this issue (*ε*_clonogenic_ = 4.8 · 10^−4^)—suggesting the use of a joint approach where data from both assays is used. Bottom: experimental and synthetic comet readout for the same parameter sets.

Approximate Bayesian computation (ABC) [37] is used to estimate distributions of model parameters. The method works as follows: for each parameter, the range of biologically meaningful parameter values is estimated. For example, with *γ* = 10 h ^−1^ (the upper boundary of this parameter), repair proteins reactivate very quickly from the irradiation event; the repair probability recovers to 94% 30 min post irradiation. This is unrealistically high given the typical delays observed experimentally (see Figure 4 and [38]). Since no prior information is available on a given parameter values' positions within the search space [*a*; *b*], uniform prior distributions with boundaries *a* and *b*, *𝒰*[*a*; *b*], are chosen. The boundaries are listed in Table 1 for each parameter. Determining the lower bound on *α* presents a special case: it is easy to show that in the absence of any repair (i.e., *r*(*H*_1_) = 0),

(9) $$\begin{array}{c} H_{0}\left(t\right)=\exp\left(-\alpha Rt\right), \end{array}$$

for the duration of irradiation. After irradiation, *R* = 0, and thus, *H*_0_(*t*) remains constant. Because *H*_0_(*t*) is mapped to the fraction of surviving cells (see Section 2.5), a lower bound for *α* can be established by solving Equation (9) after substituting *H*_0_(*t*) for *S* as reported in the clonogenic assay and setting *t* to the point in time at which irradiation ends.

At the beginning of the parameter search, *n* sets of parameters are initialized by drawing from the prior distributions. Predictions are made by running the model in a forward fashion, extracting the predicted readout as described in Section 2.5 and comparing it to experimental data. This yields an error *ε* according to Equations (10) and (11) (see below).

In each iteration of the search, the parameters are perturbed; the new parameter values are kept if *ε* decreases and are discarded otherwise. In a simulated annealing fashion [39], the amount of perturbation is gradually decreased as the search progresses. A cut-off value of *ε* = 10^−2^ was chosen. In the end, *n* sets of parameters are left; all of which provide a satisfactory error. In this work, *n* = 1000 was chosen with 250 iterations.

The objective function for the parameter search with cell survival data is

(10) $$\begin{array}{c} \varepsilon_{\text{clonogenic}}=\underset{\text{D}}{\sum }\;{\left(\log\left(S_{D}\right)-\log\left({\overset{\wedge }{S}}_{D}\right)\right)}^{2}, \end{array}$$

for the radiation doses *D*, the experimentally obtained surviving fraction of cells *S*_*D*_, and the predicted surviving fraction of cells $\hat{S}$.

Similarly, the objective function for the parameter search with comet data is

(11) $$\begin{array}{c} \varepsilon_{\text{comet}}=\underset{t>0}{\sum }\;\overset{K}{\underset{i=0}{\sum }}\;{\left({\tilde{h}}_{i}^{\left(t\right)}-{\tilde{H}}_{i}\left(t\right)\right)}^{2}, \end{array}$$

for time point *t*, normalized population ${\tilde{H}}_{i}$, and normalized comet readout ${\tilde{h}}_{i}$ as defined in Equation (8). A combined calibration was attempted with a combined objective function (see discussion in Section 4).

### 2.7. Software

**Figure 5:**
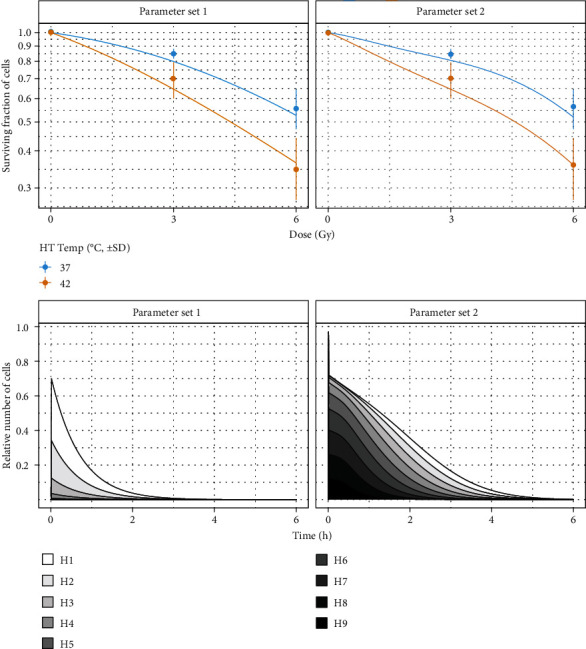
Top: cell survival curves for two different sets of parameters after parameter search with clonogenic assay data. Two sets with similar errors were chosen (*ε*_clonogenic_ = 2.5 · 10^−3^); the curves match experimental data (dots) relatively well and are very similar albeit having vastly different parameter values. Bottom: corresponding *H*_*i*_ values in time (no hyperthermia). While the two parameter sets produce similar survival curves, the dynamics of repair are very different. Because clonogenic assay captures only the state after repair has completed, no information about these dynamics is in the data. The parameters are *α* = 1.32, *c*_*r*_ = 6.89, *c*_*e*_ = 0.25, *μ*_Γ_ = 1.60, *γ* = 6.75, *a* = 0.92, *k*_2_ = 0.048, and *μ*_*Λ*_ = 1.39 (set 1) and *α* = 0.20, *c*_*r*_ = 4.58, *c*_*e*_ = 1.31, *μ*_Γ_ = 0.25, *γ* = 0.20, *a* = 0.38, *k*_2_ = 0.039, and *μ*_*Λ*_ = 3.20 (set 2).

The methods discussed above were implemented in python (version 3.5.2) using the abcpy module [40] for ABC (version 0.5.3). R version 3.6.0 was used to create the plots; the code and data are available online (https://github.engineering.zhaw.ch/weyl/synthetic_comet). The software can be configured to use either input data from clonogenic assay or input data from comet assay. Depending on this selection, the corresponding objective function *ε*_clonogenic_ or *ε*_comet_ is used. The results shown in Figure 5 are obtained with the software running on clonogenic mode, i.e., evaluating *ε*_clonogenic_, while those in Figure 4 are obtained with the software running in comet mode, i.e., evaluating *ε*_comet_.

## 3. Results

Two model outputs for cell survival are shown at the top of Figure 5, as produced by the software running in clonogenic mode. The examples were chosen according to similar *ε*_clonogenic_: for both instances, *ε*_clonogenic_ ≈ 2.5 × 10^−3^. Experimentally, it would be very challenging (if not impossible) to discriminate between the two curves. Yet, the parameters and the dynamics shown at the bottom are very different from each other: in the left case, most hits have vanished after 2 h, while the same requires 4 h in the right case.

With the software running in comet mode (i.e., minimizing *ε*_comet_), results are shown in Figure 4. The data on the left represents a random pick from the parameter result set and produces a cell survival curve very different from cell survival found experimentally (*ε*_clonogenic_=0.12). The ones on the right is the curve with the lowest error found in the set (*ε*_clonogenic_ = 4.8 · 10^−4^).

**Figure 6:**
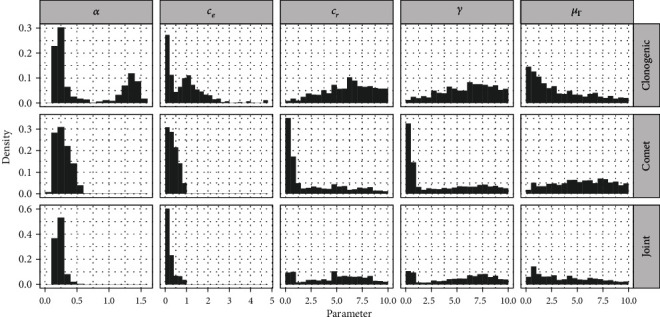
Histograms of parameter values after calibration with the software set to different modes, and at the bottom, the joint distribution obtained by combining the two posterior sets is shown. Of *n* = 1000 parameter sets, the 75% with the smallest error *ε*_clonogenic_ is used to restrict outliers.

Figure 6 shows a histogram panel of the parameters unrelated to hyperthermia (see Figure S2 for parameters *a*, *k*_2_, and *μ*_*Λ*_). In the top row, parameters from the software in clonogenic mode are shown while in the middle row, parameters from the software in comet mode are shown. The bottom row shows the joint distribution, calculated from the previous two rows. Generally, values for *α* and *c*_*e*_ are centered around one or two peaks, while, e.g., *μ*_Γ_ is more uniformly distributed in the comet case, but clonogenic assay data suggests that the parameter peaks at low values.

In addition to joining the two posterior distributions for each parameter, a calibration was attempted where the two objective functions were combined with a weighting factor *ξ*:

(12) $$\begin{array}{c} \varepsilon_{\text{combined}}=\varepsilon_{\text{clonogenic}}+\xi \varepsilon_{\text{comet}} \end{array}$$

In order not to prefer any assay source from the other, errors from the previous single-assay runs were used to select *ξ* = 1.68 × 10^−3^ such that the two terms are of the same order of magnitude. This attempt failed; the ABC solver never left its seeding state (see discussion in Section 4).

## 4. Discussion

The results shown in the previous section clearly call for a combined approach, where both clonogenic assay and comet assay data are used as sources of information for parameter search. However, the traditional approach of combining two objective functions failed. This is because in the seeding state, ABC with simulated annealing rejects samples from the prior that are above a certain threshold (values up to *ε* = 10 were tried). Since it is difficult to find parameters that satisfy both objective functions, the seeding state never completed. Thus, the computationally much lighter approach with joint posteriors is proposed, allowing for additional flexibility in combining further calibration results.

The results in Figure 5 reveal that survival curves lack sufficient information for MHR model calibration. This is hardly surprising, as it was argued before that the clonogenic assay captures information very distant from the process that is being modeled. On a side-note, any attempt to calibrate a model with 8 parameters from 5 data points is likely going to fail, which is yet another reason to include additional data sources. However, even with this little information, the top row in Figure 6 reveals regions of interest for some parameters, e.g., for *α* and *c*_*e*_. On a related note, the resulting posterior distributions for *α* and *c*_*e*_ are bimodal. This is an important finding that is concealed by a method aiming at point-estimates, such as differential evolution one used in [27]. Indeed for the *α* value, one peak of the histogram corresponds to the range of parameters found in [12], while the other peak corresponds to the range of parameters found in [27]. Interestingly, these two regimes also correspond to the two instances depicted in Figure 5.

Based on the aforementioned rationale, one may assume that the use of comet assay readouts would cure these issues. However, Figure 4 demonstrates that this is not the case. Otherwise, any parameter set would yield an adequate cell survival curve. Discussing potential explanations for this observation is critical since the resulting conclusions govern the choice of further data to address the open issues: for quantification of the damage, the relative tail intensity is assessed for approx. 100 cells per assay. This quantification does not discriminate between cells that have a chance to reach *ℋ*_0_, cells that have already initiated apoptosis and will never reach *ℋ*_0_, and cells that are on the brink of death for other reasons. In fact, the quantification may even contain cells that are already dead but still have DNA that is visible in the microscopy image. However, the ability to reach *ℋ*_0_ is essential for producing a survival curve from the model. Thus, a possible explanation for the inability to achieve successful model calibration from comet assay readout alone could be that the readout does not carry sufficient information about the ability to reach *ℋ*_0_. Furthermore, dead cells that have degraded so far as to not have any quantifiable DNA whatsoever would not be considered for comet assay, and the normalization of the 100 cells to a relative histogram would mostly masquerade their existence: the only way for dead cells to influence the results is in the ratio ${\tilde{h}}_{0}/\sum_{i\neq 0}\;{\tilde{h}}_{i}$, since a surviving cell would contribute to *H*_0_ (thus increasing *H*_0_), but if the same cell had died, it would not contribute to any *H*_*i*_ (thus increasing ${\tilde{H}}_{i}$ for *i* ≠ 0).

For the parameters *c*_*r*_, *γ*, and *μ*_Γ_ (Figure 6) as well as the parameters related to hyperthermia (Figure S2), uniform posterior distributions are obtained. The method thus reveals that more input data is required to identify these parameters. Parameters *γ* and *μ*_Γ_ relate to a transient repair inability due to the irradiation event. From Figure 4, it can be seen that this effect vanishes approx. 30 min. after irradiation. Thus, further data within that time frame could yield better estimates for those parameters. *c*_*r*_ could be identified by running a series of clonogenic assays at various dose rates. At low dose rates, irradiation would not be considered as an event but have a finite duration and repair may start already during irradiation. Such dose-rate-dependency was shown in the past to be reproduced by the MHR model [12], and the rate of repair *c*_*r*_ could become identifiable. The hyperthermia parameters could be refined with data from a study with varying time-gaps. Such data from clonogenic assay has been publishedI [9] but not from comet assay and with different cell lines. As mentioned in Introduction, assessing the repair-protein reactivation rate constant *k*_2_ would be of great clinical use, as it would allow a better assessment about tolerable time-gaps (and variation thereof) between irradiation and hyperthermia. Since the order of the two treatments (i.e., hyperthermia prior to versus after irradiation) was shown to have minimal effect on cell survival [9], additional input data in this regard would likely not improve the calibration results. Investigating more cell lines would reveal which parameters may vary by how much between subjects.

Clonogenic cell survival and comet assay measurements were shown to be repeatable [14, 29]; thus, it is reasonable to expect repeatable results from patient biopsies [38]. This would allow for a per-patient calibration, e.g., to improve the treatment plan on a per-patient basis. As mentioned in the previous paragraph, such an endeavour would require data from appropriate sources to identify the relevant parameters, rather than just more amounts of data.

In case the model cannot be calibrated at all despite these efforts, it could be simplified, for example by replacing the TBDE Γ with a fixed window of no repair after irradiation, removing the parameters *μ*_Γ_ and *γ*. Alternatively, it is conceivable to split the process at the time of the irradiation, yielding a hyperthermia process that sets up the initial conditions for a subsequent DNA damage and repair process. Splitting the model in this way could yield closed-form solutions or approximations thereof for some state variables, paving the way for a much simpler calibration strategy.

The model and the strategy presented in this work have a number of potential limitations. First, the model does not incorporate any mitosis, which occurs without doubt in *ℋ*_0_ until the cells are fixed and the clonogenic assay is performed. However, one can argue that for a given cell line, any mitosis would occur at a fixed rate. While the number of cells would increase, their ratio would remain the same. Because the clonogenic cell survival assays used in this work are normalized, mitosis cancels out. Some cells may, however die only after a few cell cycles. This falls in the gap between the last comet assay and the point in time when clonogenic assay is performed and is not modeled in the MHR model. Second, the model does not incorporate any effects of direct cytotoxicity, i.e. thermal cell-killing. This is alleviated by the fact that such direct cytotoxicity was not observed in any of the control experiments performed with hyperthermia alone [29]. Third, the model does not correctly describe inhibition of DNA repair proteins above a temperature threshold of 42.5°C–43°C, since those proteins are believed to enter a different regime above that threshold [35]. While this is a limitation, it does not affect the work presented here since the highest temperature applied *in vivo* and in silico was 42°C.

## 5. Conclusions

This work demonstrates that a holistic approach is necessary to calibrate the MHR model parameters. Relying on clonogenic assay data or comet assay data alone, as it has been done in the past, proved to be insufficient to establish unambiguous model parameters. Even with this combined approach, some parameters remain unidentified. However, the ABC method has the advantage of joining existing posterior distributions with distributions obtained from calibration runs with new input data. This ability is critical since model calibration with ABC is, despite all its advantages, very slow. Combining posterior distributions from ABC, however, is fast. Following this approach, data from different assays can be combined in a modular fashion without the need of rerunning the full calibration.

While the application of the method presented is radiotherapy, hyperthermia, and treatment planning, the method presented here addresses a more general problem; thus, many other instances exist where the application of this method would be of value.

## Figures and Tables

**Table 1:** Summary of model parameters including their search space. The last column indicates the result of parameter search (see Section 2.6).

Parameter	Description	Search space
*α*	Radiosensitivity (Gy ^−1^)	[0.17; 2]
*c*_*r*_	Repair rate constant (h ^−1^)	[0; 10]
*c*_*e*_	Elimination rate constant (h ^−1^)	[0; 10]
*μ*_Γ_	TBDE weighting factor (Gy ^−1^)	[0; 10]
*γ*	TBDE rate constant (h ^−1^)	[0; 10]
*a*	Repair protein deactivation rate (h ^−1^)	[0; 2]
*k*_2_	Repair protein activation rate (h ^−1^)	[0; 0.1]
*μ*_*Λ*_	Hyperthermia weighing factor	[0; 5]

## Data Availability

The data are available online (https://github.engineering.zhaw.ch/weyl/synthetic_comet) along with the software code required to perform the analyses shown in this work.

## Supplementary Materials

Supplementary Materials The supplementary materials contain (A) the full equation system of the multi-hit-repair (MHR) model and (B) supplementary figures with additional parameter histograms.
