# Supplementary material for: Holistic View on Cell Survival and DNA Damage: How Model-Based Data Analysis Supports Exploration of Dynamics in Biological Systems
Source: Comput Math Methods Med. 2020 Jul 6;2020:5972594. doi: 10.1155/2020/5972594 (PMC7361897; doi:10.1155/2020/5972594)
Supplement: Supplementary Materials — The supplementary materials contain (A) the full equation system of the multi-hit-repair (MHR) model and (B) supplementary figures with additional parameter histograms. [file 5972594.f1.pdf]

A. The MHR Model

The MHR model system is defined as follows:

$$
\begin{aligned}
\frac{\mathrm{d}H_0}{\mathrm{d}t} &= -\alpha R H_0 + r(H_1) \\
\frac{\mathrm{d}H_1}{\mathrm{d}t} &= \alpha R H_0 - \alpha R H_1 - r(H_1) - c_e H_1 + r(H_2) \\
&\cdots \\
\frac{\mathrm{d}H_{i-1}}{\mathrm{d}t} &= \alpha R H_{i-2} - \alpha R H_{i-1} - r(H_{i-1}) - c_e H_{i-1} + r(H_i) \\
\frac{\mathrm{d}H_i}{\mathrm{d}t} &= \alpha R H_{i-1} - \alpha R H_i - r(H_i) - c_e H_i + r(H_{i+1}) \\
\frac{\mathrm{d}H_{i+1}}{\mathrm{d}t} &= \alpha R H_i - \alpha R H_{i+1} - r(H_{i+1}) - c_e H_{i+1} + r(H_{i+2}) \\
&\cdots \\
\frac{\mathrm{d}H_K}{\mathrm{d}t} &= \alpha R H_{K-1} - r(H_K) - c_e H_K \\
\frac{\mathrm{d}\Gamma}{\mathrm{d}t} &= R - \gamma\Gamma \\
\frac{\mathrm{d}\Upsilon}{\mathrm{d}t} &= -k_1\Upsilon + k_2\Lambda \\
\frac{\mathrm{d}\Lambda}{\mathrm{d}t} &= k_1\Upsilon - k_2\Lambda \\
r(H_i) &= c_r \exp\left(-\mu_\Gamma\Gamma - \mu_\Lambda\Lambda\right) H_i \\
k_1 &= a \cdot 10^{-3} \exp\left(\frac{E_a}{\bar{R}(273.16 + 37)} - \frac{E_a}{\bar{R}(273.16 + T)}\right)
\end{aligned}
$$

$\bar{R}$ is the gas constant, $E_a = 1528$ kJ·mol^{-1} is the activation energy, $T(t)$ is the temperature in °C, and $R(t)$ is the dose rate of radiation administered.

B. Supplementary Figures

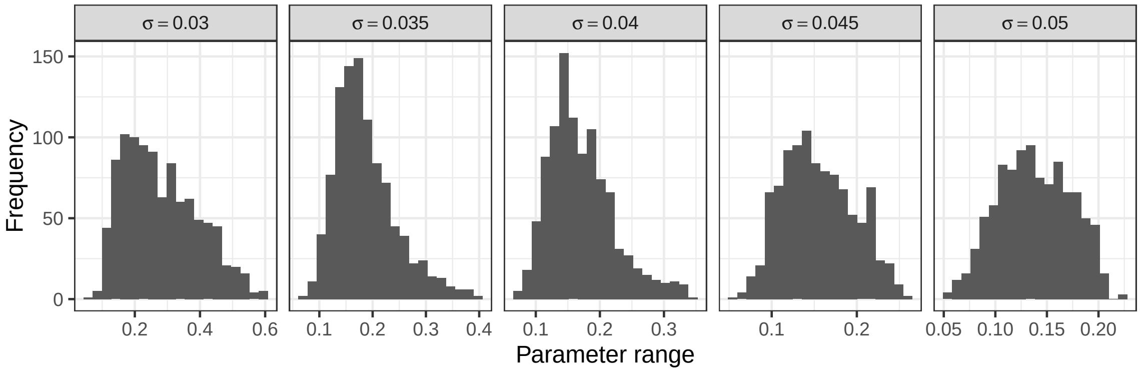

Figure S1: Histograms for α after minimizing ϵ_{comet} with different values for σ. For $\sigma > 0.03$, most values of α exceed the lower boundary of 0.17 Gy^{-1} stipulated by Eq. 9. The calibrations for these plots were done with a lower bound of $\alpha_{\text{min}} = 0$ Gy^{-1} in order to demonstrate this issue.

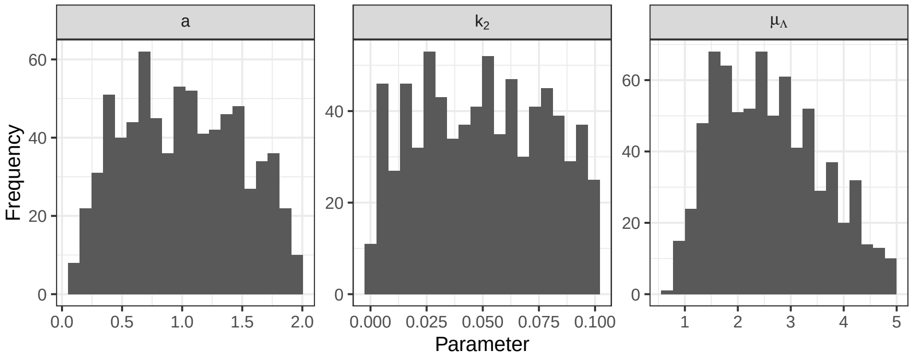

Figure S2: Histograms for a, k_2 and μ_Λ after calibration in clonogenic-mode. The parameters cover the search space almmost uniformly, suggesting that ambiguities in these parameters remain.
